# Pulmonary epithelioid hemangioendothelioma: a case report

**DOI:** 10.4076/1757-1626-2-8235

**Published:** 2009-09-03

**Authors:** Yassine Ouadnouni, Mohammed Bouchikh, Abdellah Achir, Fouad Zouaidia, Mohammed Smahi, Yassine Msougar, Marouane Lakranbi, Said Afqir, Najat Mahassini, Abdellatif Benosman

**Affiliations:** 1 Department of Thoracic Surgery, Ibn Sina University HospitalRabatMorocco; 2 Department of Anatomical Pathology, Ibn Sina University HospitalRabatMorocco; 3 Department of Medical Oncology at National Institute of OncologyRabatMorocco; 4 Department of Thoracic Surgery, Hassan II University HospitalFesMorocco

## Abstract

**Introduction:**

The pulmonary epithelioid hemangioendothelioma is a rare vascular intermediate malignancy tumour.

**Case presentation:**

A 45-year-old man, he shows an isolated chronic cough with a preserved general state of health. The thoracoabdominal Computed tomography showed three well limited opacities of the right lung, among them one shows some calcifications; which we entirely resected by enucleation after a pneumotomy. The histologic examination with immunomarking led to an epithelioid hemangioendothelioma.

**Conclusions:**

The pulmonary epithelioid hemangioendothelioma is a tumour of unpredictable prognosis, bad when linked to the plurifocal and symptomatic forms.

## Introduction

The pulmonary epithelioid hemangioendothelioma is a rare vascular intermediate malignancy tumour. The surgical treatment is often disapproved in relation with the multicentric character of the lesions. We report the observation of a multifocal form treated surgically, with a favourable development.

## Case presentation

**Figure 1.**
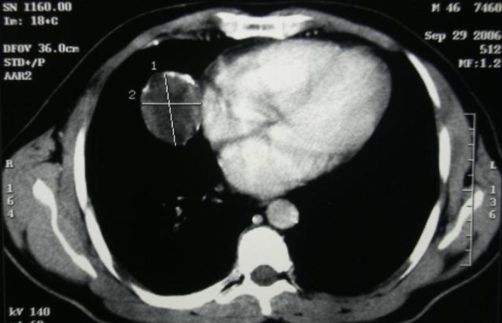
Computed tomography scan showing tissular mass with peripheral calcification in the right lung.

**Figure 2.**
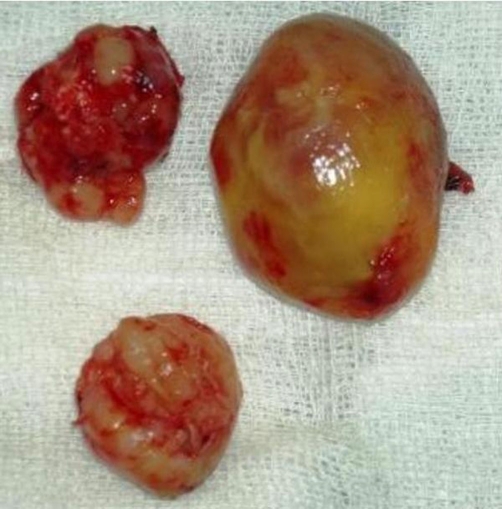
Surgical specimen.

**Figure 3.**
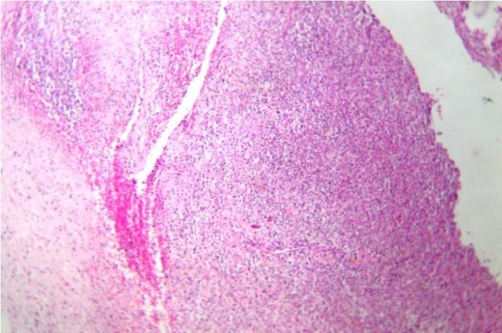
Photomicrograph showing tumoral proliferation with vascular spaces (hematoxylin-eosin stain ×10).

**Figure 4.**
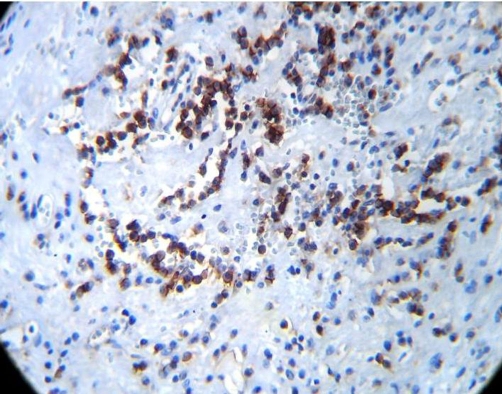
Photomicrograph showing tumoral cells immunomarquing with anti CD 31 antibody ×40.

A 45-year-old man, African origin and Moroccan nationality, with no specific history. He shows an isolated chronic cough with a preserved general state of health. The clinical and biological examinations were normal. The thoracic radiograph showed three well limited opacities of the right lung, among them one contained some calcifications. The thoracoabdominal Computed tomography has clarified the tissular density with neither other mediastinal nor abdominal localisations (Figure 1). The bronchial fibroscopy was normal. A right posterolateral thoracotomy showed three solid masses, each of them located in a lobe, which we entirely resected by enucleation after a pneumotomy (Figure 2). The histologic examination with immunomarking led to an epithelioid hemangioendothelioma (Figures 3 and 4). The development was favourable after a thirty-month follow-up.

## Discussion

The epithelioid hemangioendothelioma is a rare vascular tumour resulting from endothelial cells. Dail and Liebow [1], described the pulmonary attack in 1975, regarded initially as a variant of bronchioalveolar carcinoma called Intravascular Bronchioloalveolar Tumour. The epithelioid hemangioendothelioma affects, with predilection, the limp tissues. The most frequent visceral affection is hepatic. Nevertheless, the pulmonary localisation remains rare [2]. Actually, the pulmonary epithelioid hemangioendothelioma has only been reported in about a hundred cases since the description of the disease. It affects preferentially the young adult and twice more women than men. The etiological factors are not well known. However, the presence of the estrogenic receptors in an inconstant way in the tumour and the occurrence in man gender, do not support the hormonal hypothesis. The pulmonary radiological signs are not specific. They often correspond to nodular lesions disseminated in less than 2 cm, sometimes calcified, either unilateral in almost 20% of cases, or bilateral giving an aspect of a balloons’ release in about 60% of cases. An isolated pulmonary mass of less than 5 cm is noticed in 10% of the cases [3]. Our patient presents some multifocal and unilateral nodular radiological images with a mass exceeding 3 cm, which associates two kinds of radiological aspects of this exceptional tumour. The bronchial fibroscopy is more often normal, except from a diffuse bleeding at the time of disseminated forms. The bronchial biopsies and the bronchioalveolar lavage are not contributory. The diagnosis is generally done on a surgical pulmonary biopsy. The histology is characteristic: macroscopically, it corresponds to an aspect of cartilaginous spherical lesions, translucent during the splits up, with a necrosed center. The optical microscope objectivises mononucleated cells of large sizes and an abundant eosinophilic cytoplasm. The immunohistochemistry confirms the diagnosis with a positive immunomarking of antibodies anti-CD31, anti-factor VIII and anti-CD34 [3]. The tumour can be confined to the lung or associated to other localisations. These secondary localisations do not have the same signification as in the lung cancer, on account of the possibility of a primitive multifocal attack (multicentric eclosion disease) [3]. In our case, the affection is multifocal and limited to the right lung. The treatment of the epithelioid hemangioendothelioma is not well codified, the therapeutic abstention is recommended in the asymptomatic pulmonary localisation. The surgery is suggested in case of single nodule or unilateral multiples. However, it does not seem to be effective in the bilateral multiple forms [4]. In the extended forms, a neoadjuvant or adjuvant therapy to surgery (corticotherapy, chemotherapy and radiotherapy) showed a partial efficiency. In our case the surgical resection was considered satisfying, we did not use a complementary treatment [5,6].

In general, during the pulmonary epithelioid hemangioendothelioma, we notice two population groups:

A first asymptomatic group or a few with radiological images: an isolated nodular or unilateral multiple. These tumours are prepared to a programmed surgery or atypical resections with a systematic ganglionic curage even if a lymph invasion is unlikely (less than 9%). A survival median is estimated to more than 10 years.

A second symptomatic group with, at the radiology, an infiltrative affection of the parenchyma or a pleural effusion, responding to polychemotherapy. The prognosis remains severe.

The factors of a bad pulmonary epithelioid hemangioendothelioma prognosis are: the presence of a respiratory symptomatology, a young age, a multifocal affection; Histologically, a mass more than 3 cm, and a high mitotic activity as well as cytonuclear atypicality [4]. Our observation is particular on account of the tumour’s pulmonary multifocal localisation, with a mass exceeding 3 cm with a favourable development after only a surgical treatment.

## Conclusions

The pulmonary epithelioid hemangioendothelioma is a tumour of unpredictable prognosis, bad when linked to the plurifocal and symptomatic forms. The diagnosis is histologic, requiring an immunohistochemical study. The surgery is indicated for unilateral nodular affections whether unique or multiple. The bilateral localisations are the concern of the polychemotherapy, whose protocol is not codified yet, and the results less satisfying.
